# Supplementary material for: Pre-Existing Immunity Predicts Response to First-Line Immunotherapy in Non-Small Cell Lung Cancer Patients
Source: Cancers (Basel). 2024 Jun 28;16(13):2393. doi: 10.3390/cancers16132393 (PMC11240823; doi:10.3390/cancers16132393)
Supplement: Supplementary file 1 [file cancers-16-02393-s001.zip › cancers-3051076-supplementary.pdf]

Supplemetrary Materials

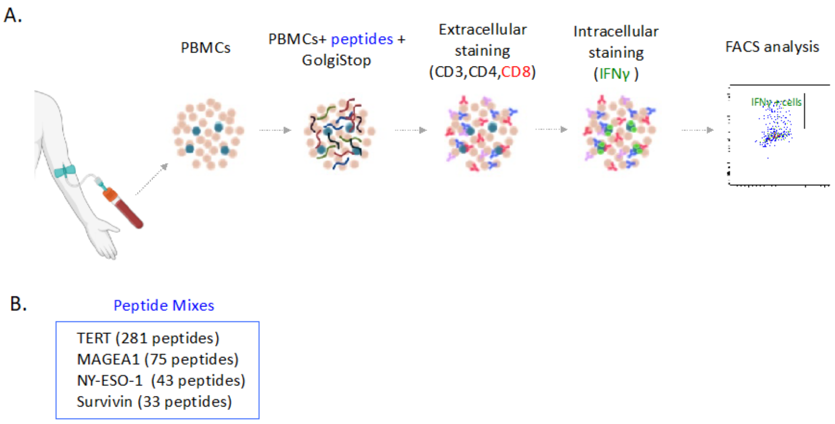

Figure S1. Experimental flow chart for Pre-existing Immunity detection.

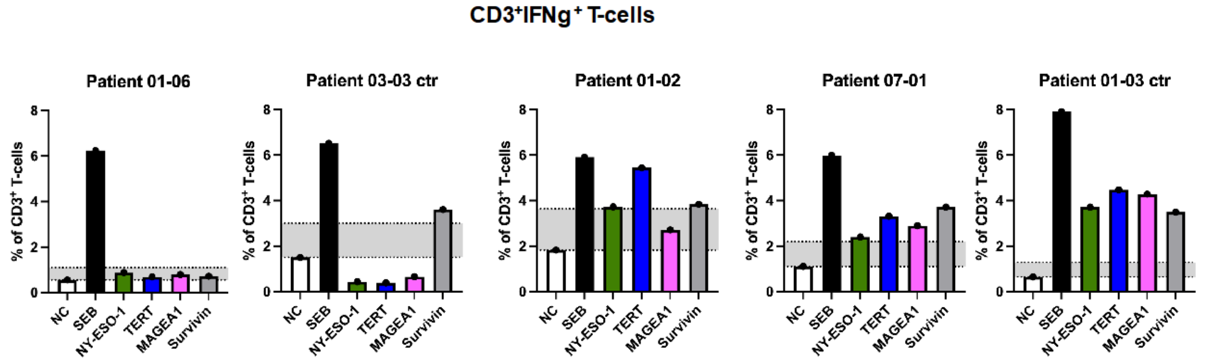

Figure S2. Persentages of $CD3^+IFNg^+$ T-cells (PreI+ T-cells) in 5 representative patients of the study. Grey box represents 2-fold induction over NC; NC: cells treated with DMSO ; SEB: cells treated with Staphylococcal enterotoxin B. Graps represent the average persentage of IFNg + T-cell of 3 technical replicates for each patient.

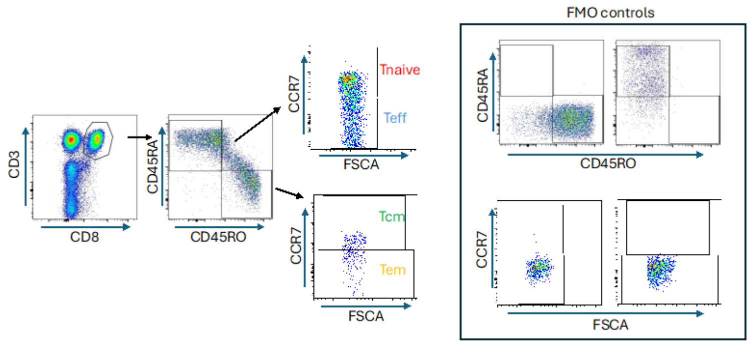

Figure S3. Gating strategy for different subtypes of $CD8^+$ T-cells. FMO controls: fluoresent minous one ; Tnaive : $CD3^+CD8^+$ $CD45RA^+CD45RO^-CCR7^+$; Teff: : : $CD3^+CD8^+$ $CD45RA^+CD45RO^-CCR7^-$; Tcm: : $CD3^+CD8^+$ $CD45RA^-CD45RO^+CCR7^+$; Tem: : $CD3^+CD8^+$ $CD45RA^-CD45RO^+CCR7^{-}$.

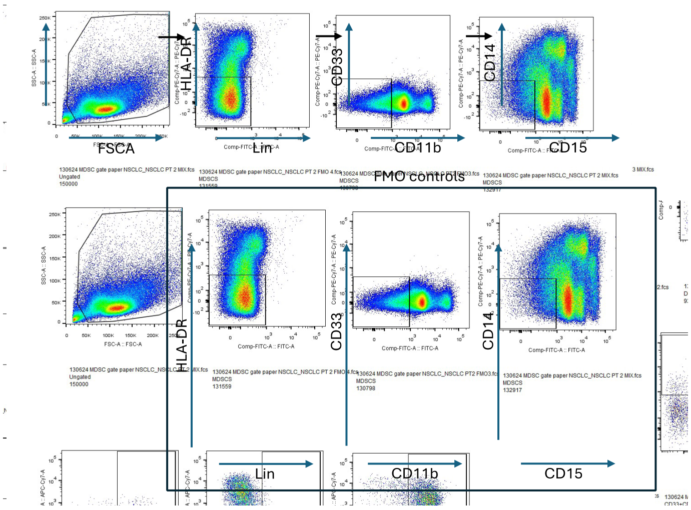

Figure S4. Gating strategy for MDSCs. Cells are first gated to exclude cell debris on SSC and FSC. Then the vibrable cells are gated on the absence of HLA-DR and Lin expression. Next MDSCs are defined by their expression of CD33 and CD11b. At the end M-MDSCs are defied as CD14^{+} CD15^{-} or CD14^{+}CD15^{+}. FMO controls: fluoresent minous one.

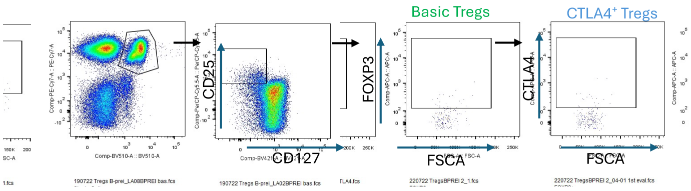

Figure S5. Gating strategy for Tregs.

Table S1. Percentages of different clinical characteristics in patients with or without pre-existing immunity.

	% of patients		
	Total	PreI-	PreI+
**Gender**			
Male	77% (40/52)	55% (22/40)	45% (18/40)
Female	23% (12/52)	58% (7/12)	42% (5/12)
**Histology**			
Adenocarcinoma	50% (26/52)	54% (14/26)	46% (12/26)
Squamous	46% (24/52)	54% (13/24)	46% (11/24)
Unknown	4% (2/52)	100% (2/2)	0% (0/2)
**Location of primary tumor**			
Left lung	31% (16/52)	63% (10/16)	37% (6/16)
Right lung	62% (32/52)	50% (16/32)	50% (16/32)
Both lungs	3.5% (2/52)	50% (1/2)	50% (1/2)
Unknown	3.5% (2/52)	100% (2/2)	0% (0/2)
**Age**			
<70	46% (24/52)	54% (13/24)	46% (11/24)
>70	50% (26/52)	54% (14/26)	46% (12/26)
Unknown	4% (2/52)	100% (2/2)	0% (0/2)
**Smoking status**			
Never	8% (4/52)	50% (2/4)	50% (2/4)
Former	44% (23/52)	52% (12/23)	48% (11/23)
Curent	40% (21/52)	52% (11/21)	48% (10/21)
Unknown	8% (4/52)	100% (4/4)	0% (0/4)
< 40 pack year	17% (9/52)	55% (5/9)	45% (4/9)
40-80 pack year	29% (15/52)	33% (5/15)	67% (10/15)
> 80 pack year	19% (10/52)	40% (4/10)	60% (6/10)
Unknown	35% (18/52)	55% (10/18)	45% (8/18)

Table S2. Cox regression analysis.

Risk Factor	Parameter Estimate	P-Value	Hazard Ratio (HR) (95% CI for HR)
Age, years	0.967	0.204	"0.9184 to 1.018"
Sex, male	0.963	0.935	"0.4087 to 2.645"
Smoking, former	2.044	0.265	"0.4680 to 6.355"
Smoking, curent	0.895	0.790	"0.3972 to 2.058"
Hystology	1.943	0.109	"0.8709 to 4.531"
Location	0.969	0.940	"0.4123 to 2.125"
Smoking (py)	0.610	0.447	"0.1555 to 2.086"

Table S3. Percentages of different immune cell populations between patients with and without pre-existing immunity.

			% in CD3CD8 T-cells		% in CD3CD4 T-cells	
			PreI -	PreI +	PreI -	PreI +
	RA+RO-CCR7+	Mean	34.89	41.04	75.2	75.28
		Std.Error	4.25	4.61	4.09	3.45
		p-value	0.256		0.562	
	RA+RO-CCR7-	Mean	62.9	57.01	25.68	23.64
		Std.Error	4.40	4.22	3.67	4.11
		p-value	0.237		0.783	
	RA-RO+CCR7+	Mean	47.56	48.43	57.68	50.44
		Std.Error	4.71	4.14	4.48	4.47
		p-value	0.767		0.279	
	RA-RO+CCR7-	Mean	50.49	50.18	40.20	49.40
		Std.Error	4.60	4.21	4.19	4.66
		p-value	0.843		0.168	
PD-1+	RA+RO-CCR7+	Mean	4.17	5.37	17.27	10.18
		Std.Error	0.72	1.95	2.97	1.65
		p-value	0.848		0.132	
	RA+RO-CCR7-	Mean	4.36	3.89	7.32	6.46
		Std.Error	0.57	1.02	1.50	1.09
		p-value	0.179		0.992	
	RA-RO+CCR7+	Mean	36.40	32.62	9.42	7.71
		Std.Error	3.18	2.74	1.25	1.00
		p-value	0.605		0.535	
	RA-RO+CCR7-	Mean	30.58	28.37	8.40	8.61
		Std.Error	2.23	2.95	0.94	1.35
		p-value	0.242		0.790	

Table S4. Percentages of different immune cell populations between patients and healthy donors.

	% T-cell populations		NSCLC	HD
% in CD3+CD8+	Tnaive (RA+RO-CCR7+)	Mean	15.48	19.98
		p-value	0.423	
	Teff (RA+RO-CCR7-)	Mean	16,51	8,32
		p-value	0,175	
	Tcm (RA-RO+CCR7+)	Mean	17.6	16.63
		p-value	0.760	
	Tem (RA-RO+CCR7-)	Mean	11.41	11.02
		p-value	0.908	
% in CD3+CD4+	Tnaive (RA+RO-CCR7+)	Mean	8.075	8.93
		p-value	0.694	
	Teff (RA+RO-CCR7-)	Mean	2.023	1.524
		p-value	0.585	
	Tcm (RA-RO+CCR7+)	Mean	43.32	39.76
		p-value	0.568	
	Tem (RA-RO+CCR7-)	Mean	10.14	12.56
		p-value	0.531	
